# Metal-sensitive and thermostable trypsin from the crevalle jack (*Caranx hippos*) pyloric caeca: purification and characterization

**DOI:** 10.1186/1752-153X-7-166

**Published:** 2013-10-10

**Authors:** Helane MS Costa, Augusto CV Freitas Júnior, Ian PG Amaral, Izaura Y Hirata, Patrícia MG Paiva, Luiz B Carvalho, Vitor Oliveira, Ranilson S Bezerra

**Affiliations:** 1 Laboratório de Enzimologia (LABENZ), Departamento de Bioquímica (CCB) and Laboratório de Imunopatologia Keizo Asami (LIKA), Universidade Federal de Pernambuco, Av. Prof. Moraes Rego s/n, Cidade Universitária, Recife, Pernambuco 50670-910, Brazil; 2 Departamento de Biofísica, Escola Paulista de Medicina, Universidade Federal de São Paulo, Rua Três de Maio, 100, São Paulo 04044-020, Brazil; 3 Laboratório de Glicoproteínas, Departamento de Bioquímica (CCB), Universidade Federal de Pernambuco, Av. Prof. Moraes Rego s/n, Cidade Universitária, Recife, Pernambuco 50670-910, Brazil

**Keywords:** *Caranx hippos*, Crevalle jack, Fish trypsin, Marine fish, N-terminal amino acid sequence, Thermostable trypsin, Waste recovery

## Abstract

**Background:**

Over the past decades, the economic development and world population growth has led to increased for food demand. Increasing the fish production is considered one of the alternatives to meet the increased food demand, but the processing of fish leads to by-products such as skin, bones and viscera, a source of environmental contamination. Fish viscera have been reported as an important source of digestive proteases with interesting characteristics for biotechnological processes. Thus, the aim of this study was to purify and to characterize a trypsin from the processing by-products of crevalle jack (*Caranx hippos*) fish.

**Results:**

A 27.5 kDa trypsin with N-terminal amino acid sequence IVGGFECTPHVFAYQ was easily purified from the pyloric caeca of the crevalle jack. Its physicochemical and kinetic properties were evaluated using N-α-benzoyl-_DL_-arginine-p-nitroanilide (BApNA) as substrate. In addition, the effects of various metal ions and specific protease inhibitors on trypsin activity were determined. Optimum pH and temperature were 8.0 and 50°C, respectively. After incubation at 50°C for 30 min the enzyme lost only 20% of its activity. *K*_*m*_, *k*_*cat*,_ and *k*_*cat*_/*K*_*m*_ values using BApNA as substrate were 0.689 mM, 6.9 s^-1^, and 10 s^-1^ mM^-1^, respectively. High inhibition of trypsin activity was observed after incubation with Cd^2+^, Al^3+^, Zn^2+^, Cu^2+^, Pb^2+^, and Hg^2+^ at 1 mM, revealing high sensitivity of the enzyme to metal ions.

**Conclusions:**

Extraction of a thermostable trypsin from by-products of the fishery industry confirms the potential of these materials as an alternative source of these biomolecules. Furthermore, the results suggest that this trypsin-like enzyme presents interesting biotechnological properties for industrial applications.

## Background

Fish processing generates large quantities of liquid and solid wastes including skin, bones, fins, heads, and viscera. These by-products have no commercial value and are generally discarded without treatment, causing environmental pollution. In our laboratory, fishery by-products have been proposed as a low-cost source of biomolecules such as proteases [1-6]. These digestive enzymes constitute one of the most important groups of industrial enzymes, with applications in a wide variety of industries including the detergent, food, agrochemical, and pharmaceutical industries, and account for at least 60% of all global enzyme sales [7,8]. Trypsins (EC 3.4.21.4) are present in the digestive tract of fish and have largely identical substrate specificities [9]. These enzymes are generally thermostable, showing high activity at alkaline pH and sensitivity to numerous metal ions [1-4,6,9-13].

Crevalle jack (*Caranx hippos*) is a marine fish found in tropical and subtropical zones worldwide. In the American continent, it is found from Nova Scotia to the Gulf of Mexico and Caribbean and as far south as the Uruguayan coastline [14]. This fish has significant commercial importance to the Brazilian fishery industry, with approximately 2,500 tons being captured along the Brazilian coast in 2010 [15]. As a typical carnivorous fish, it possesses a digestive tract comprising a stomach that leads to the pyloric caeca and a short intestine. High quantities of alkaline proteases in the pyloric caeca of the fish have been revealed, with trypsin found to be responsible for most of the proteolytic activity [16]. The objective of this study was to purify and partially characterize a thermostable trypsin from the pyloric caeca of *C. hippos* and determine its N-terminal sequence.

## Results and discussion

### Enzyme purification

Table 1 shows purification results. In three steps (heat treatment, ammonium sulfate precipitation, and Sephadex G-75 chromatography), an enzyme preparation was purified approximately 102-fold with an 18% yield as per protocols described to purify trypsin from other fish [1-3,6,9,17].

**Table 1:** Purification of trypsin of crevalle jack pyloric caeca

**Step**	**Total protein (mg)**	**Total activity (U)**	**Specific activity (U/mg)**	**Yield (%)**	**Purification (fold)**
Crude extract	2 040. 0	1.863	0.9	100.0	1.0
Heat treatment	1 560. 0	1.743	1.1	85.4	1.2
Ammonium sulfate precipitation (F 0–80%)	85.6	387	4.5	20.8	5.0
Sephadex G-75	3.7	341	92.2	18.3	102.4

Protein and enzyme activity were established according to Warburg and Christian [18] and Freitas-Júnior *et al*. [2], respectively.

**Figure 1:**
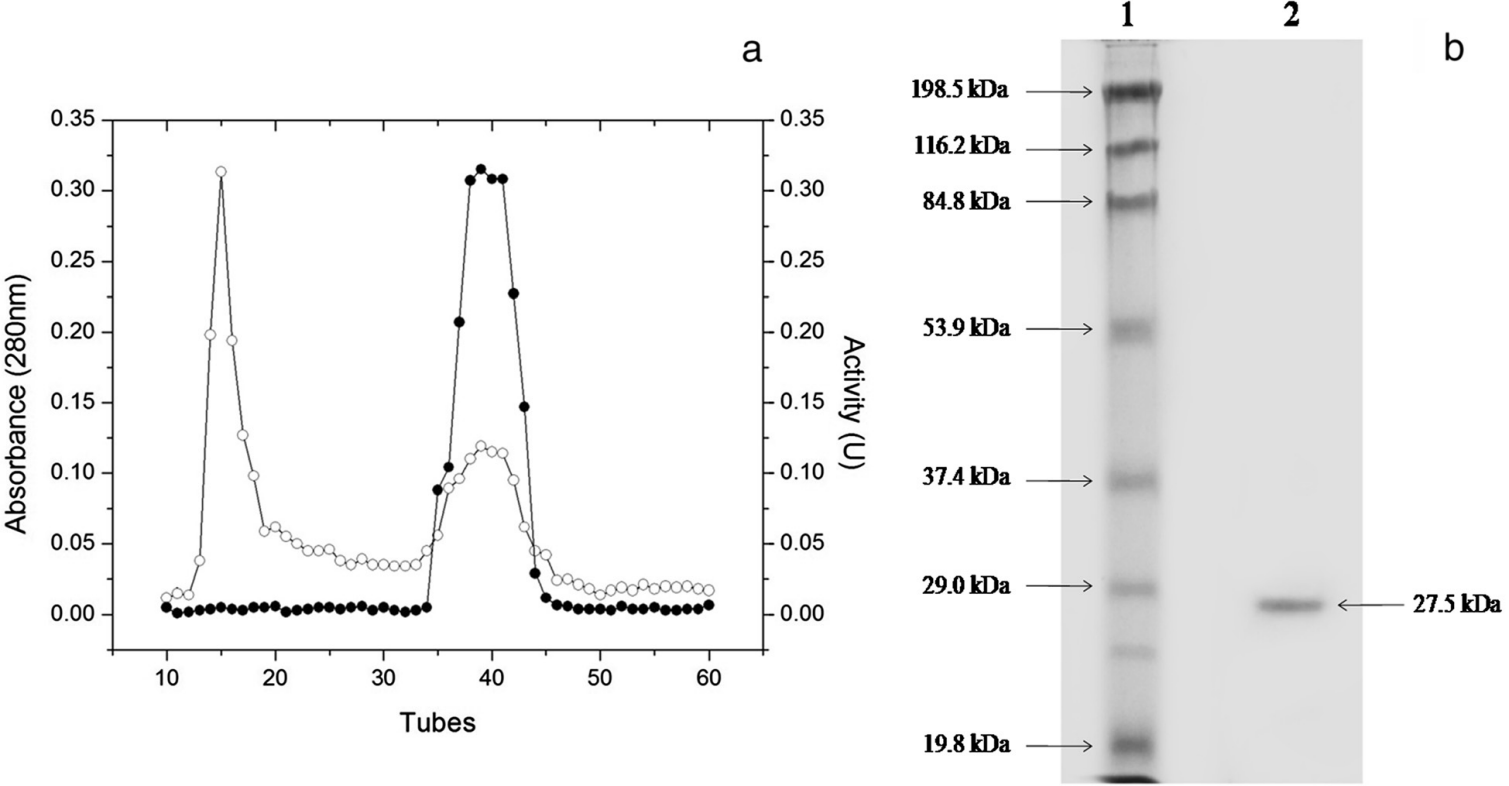
**Purification of trypsin from pyloric caeca of crevalle jack. (a)** Exclusion molecular chromatogram (Sephadex G-75) of dialyzed ammonium sulfate precipitate (F 0%–80%) obtained from crude extract of trypsin from crevalle jack: the eluted protein was monitored at 280 nm [○] and the activity (U) of each fraction was determined using BApNA as substrate [●]. **(b)** SDS-PAGE (12.5%) of crevalle jack trypsin (arrow) collected using Sephadex G-75 chromatography: (1) Molecular marker standards (myosin > β-galactosidase > bovine serum albumin > ovalbumin > carbonic anhydrase > soybean trypsin inhibitor > lysozyme); (2) crevalle jack trypsin under denaturing and reducing conditions.

The Sephadex G-75 chromatograms for trypsin from tambaqui [17], Nile tilapia [1], and spotted goatfish [6] presented profiles analogous to that found for crevalle jack (Figure 1a). A similar method using Sephadex G-100 was also used to purify trypsin-like enzymes from sardine [10], yielding identical results. Therefore, this simple and low-cost three-step method appears to present advantages over other procedures described in the literature because one of the most important limiting factors for the commercial use of fish processing waste as a protease source is the method of protein purification [6]. According to Freitas-Júnior *et al*. [2], the use of these proteases in industries, such as the food and detergent industries, does not require a high degree of purity, making the process more economically viable.

Figure 1b shows that the purified enzyme presents only one SDS-PAGE band, with a 27.5 kDa molecular mass. Fish trypsins have molecular masses between 23 and 28 kDa, e.g., *Oreochromis niloticus* (23.5 kDa) [1], *Colossoma macropomum* (23.9 kDa) [5], *Gadus macrocephalus* (24 kDa) [19], *Pseudupeneus maculatus* (24.5 kDa) [6], *Sardina pilchardus* (25 kDa) [10], *Diapterus rhombeus* (26.5 kDa) [3], *Salaria basilisca* (27 kDa) [20], *Pterygoplichthys disjunctivus* (27.5 kDa) [21], *Arapaima gigas* (28 kDa) [2], *Pomatomus saltatrix* (28 kDa) [22], and *Lutjanus synagris* (28.4 kDa) [4].

### pH and temperature effects

**Figure 2:**
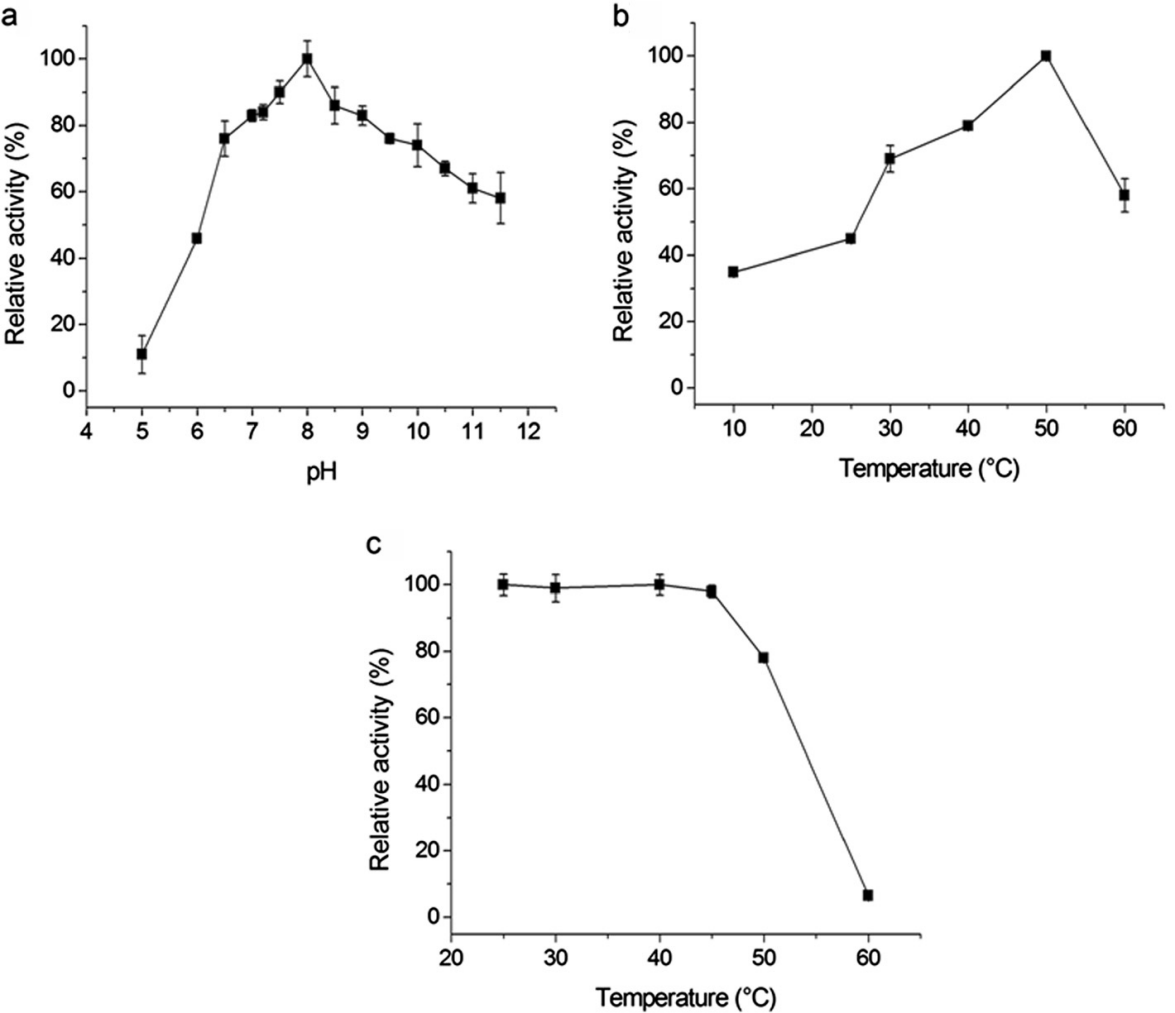
**Effects of (a) pH, (b) temperature, and (c) thermal stability on crevalle jack purified trypsin.** Samples (quadruplicate) of purified enzyme (30 μl) were assayed at pH values from 6.0 to 10.5 (Tris–HCl buffer) at temperatures ranging from 10 to 65°C. Thermal stability was determined by assaying (in quadruplicate) the enzyme activity at 25°C after pre-incubation for 30 min at temperatures ranging from 30 to 60°C.

*C. hippos* trypsin showed maximum activity at pH 8.0 (Figure 2a) and 60%–100% of this activity was achieved at a pH between 7.0 and 10.0. The loss of enzymatic activity at pH values outside the range is probably caused by protein conformational changes as a result of charge repulsion [23]. Similar behavior was observed for trypsin from Nile tilapia [1], pirarucu [2], silver mojarra [3], lane snapper [4], Monterey sardine [9], sardine [10], jacopever and elkhorn sculpin [11], skipjack tuna [12], brownstripe red snapper [13], and grey triggerfish [24] and crude extract from crevalle jack [16]. According to Maurer [25], the optimum pH is a relevant parameter that indicates the potential utilization of enzymes in detergent formulations to be used in the alkaline pH range.

The effects of temperature on trypsin activity in crevalle jack are shown in Figures 2b and 2c. Optimum temperature was found to be 50°C, supporting activity (60%–100%) over a broad temperature range (30–60°C). This result is similar to those described for trypsin from *O*. *niloticus*[1], *D*. *rhombeus*[3], *C. macropomum*[5], *Sardinops sagax caerulea*[9], *Alcichthys alcicornis*[11], *G. macrocephalus*[19], and *Theragra chalcogramma*[26]. It is noteworthy that the enzyme retained 30%–45% proteolytic activity at low temperatures (10 and 25°C), a desired property for industrial applications such as food processing operations that require low processing temperatures [27]. The enzyme maintained 100% activity at 45°C for 30 min (Figure 2c) and 80% activity at its optimum temperature (50°C). Therefore, crevalle jack protease can be considered a thermostable enzyme. Trypsins isolated from other tropical fishes showed similar behavior [1,3-6,22]. According to Gupta *et al*. [7], despite their low thermal stability above 45°C, bacterial enzymes including Alcalase, Savinase, and Esperase, (Novozymes, Denmark) as well as Maxatase (Gist-brocades, The Netherlands) are commonly used as additives in detergent. Kishimura *et al*. [26] reported a positive correlation between habitat temperature and fish trypsin thermostability. According to Freitas-Júnior *et al*. [2] the fact that some fish and aquatic organisms that live in cold waters possess digestive enzymes with high proteolytic activity at temperatures above of their habitat temperature could be related to adaptations during evolution in response to climate changes. According to Genicot *et al*. [28], thermostability and flexibility reported for several fish trypsins may be caused by structural features such as an increase in hydrophilicity and a decrease in hydrophobicity on the overall surfaces of these enzymes. Biochemical properties of crevalle jack trypsin suggest that this protease can be used in detergent formulation, and agree with those for other fish trypsins [2-5,24] because all detergent-compatible enzymes are thermally stable and alkaline with high optimum pH values [29].

### Kinetic parameters

Kinetic parameters such as the Michaelis–Menten constant (*K*_*m*_), the catalytic constant (*k*_*cat*_), and the catalytic efficiency (*k*_*cat*_/*K*_*m*_) of the purified crevalle jack enzyme were determined using BApNA as substrate (Table 2). *K*_*m*_ of the purified enzyme was 0.689 ± 0.05 mM. The *K*_*m*_ is used to assess the affinity of the enzyme for the substrate. This result indicates the considerable affinity of the purified enzyme from *C. hippos* to BApNA substrate. Similar results were found for trypsin from brownstripe red snapper (*L. vita*) [13], zebra blenny (*Salaria basilisca*) [20] and Nile tilapia (*O. niloticus*) [1]. Trypsins from other fishes [2-4,9,21,24] showed a *K*_*m*_ lower than that found for this enzyme. The *k*_*cat*_ estimates the number of substrate molecules converted into the product per second by one enzyme molecule, whereas *k*_*cat*_/*K*_*m*_ indicates the efficiency of enzyme to catalyze the transformation of substrate into product. The *k*_*cat*_ and *k*_*cat*_/*K*_*m*_ values (Table 2) showed that the enzyme was efficient in BApNA hydrolysis. The Kcat value observed for the crevalle jack trypsin shows that this enzyme converts substrate molecules into products faster than trypsin extracted from pirarucu [2], silver mojarra [3], Monterey sardine [9], brownstripe red snapper [13], zebra blenny [20] and grey triggerfish [24]. The *k*_*cat*_/*K*_*m*_ results reveal that the crevalle jack trypsin is able to hydrolyze a classic trypsin synthetic substrate more efficiently than the trypsin from *D*. *rhombeus*[3] and *A*. *gigas*[2] and similar to *L. vita*[13] and *P. disjunctivus*[21].

**Table 2:** **Kinetic parameters for trypsin from crevalle jack (*C. hippos*) using BApNA as substrate**

**Species**	**Parameters**			**References**
	***K***_**m**_**(mM)**	***k***_**cat**_**(s**^**-1**^**)**	***k***_**cat**_**/*K***_**m**_**(s**^**-1**^**mM**^**-1**^**)**	
*C*. *hippos*	0.69	6.9	10.0	Present work
*S*. *sagax c.*	0.05	2.1	41.0	[9]
*L. synagris*	0.07	-	-	[4]
*B*. *capriscus*	0.07	2.8	41.6	[24]
*P. disjunctivus*	0.13	1.46	11.24	[21]
*D*. *rhombeus*	0.27	0.9	3.48	[3]
*A*. *gigas*	0.47	1.4	2.83	[2]
*L*. *vitta*	0.51	4.7	9.27	[13]
*S. basilisca*	0.6	1.38	2.3	[20]
*O*. *niloticus*	0.77	-	-	[1]
*P*. *maculatus*	1.82	-	-	[6]

(-): data not reported.

### Effects of metal ions and inhibitors

Effects of ions and inhibitors on trypsin activity are presented in Table 3. This proteolytic activity was strongly inhibited by N-p-*tosyl*-L-*lysine* chloromethyl ketone (TLCK) and benzamidine (classic specific trypsin inhibitors) as well as phenylmethylsulphonyl fluoride (PMSF), a classic serine-protease inhibitor. The PMSF covalently binds to the serine in the enzyme active center, blocking the characteristic catalytic action of serine protease [30]. The presence of a lysine group in its structure makes TLCK a powerful trypsin inhibitor, since this inhibitory molecule covalently interacts with histidine at catalytic site blocking the enzyme active center responsible for the substrate binding [31]. According to Mihalyi [32] a real trypsin active site is inhibited by guanidines and amidines, such as benzamidine. Therefore, specific substrate and protease inhibitors provided strong evidence that trypsin was responsible for the proteolytic activity observed in the pyloric caeca of crevalle jack. Some proteins require specific metal ions as cofactors to display their biological activity. Furthermore, ionic compounds can modulate enzyme activity. The chelating agent ethylenediamine tetraacetic acid (EDTA) had no effect on trypsin activity, suggesting that this trypsin does not depend on metal ions as cofactors.

**Table 3:** Effect of ions and protease inhibitors on the trypsin of crevalle jack pyloric caeca

**Ion and inhibitor**	**Residual activity ± SD (%)**
Control*	100.0 ± 0.6^a^
**Ions (1 mM)**	
Cd^2+^	0.31 ± 0.1^b^
Al^3+^	0.42 ± 0.0^b^
Zn^2+^	17.7 ± 0.5^c^
Cu^2+^	23.8 ± 1.2^d^
Pb^2+^	38.6 ± 0.6^e^
Hg^2+^	44.9 ± 0.7^f^
Co^2+^	53.8 ± 0.1^g^
K^+^	63.3 ± 0.8^h^
Li^+^	65.4 ± 1.3^h^
Ba^2+^	67.4 ± 1.7^h^
Mn^2+^	69.4 ± 0.2^h^
Mg^2+^	75.4 ± 0.9^i^
Ca^2+^	78.9 ± 1.7^i^
**Inhibitors (1 mM)**	
PMSF	22.4 ± 1.7^b^
TLCK	0 ± 0^c^
TPCK	100.0 ± 1.2^a^
Benzamidine	1.43 ± 0.2^d^
EDTA	100.0 ± 0.7^a^

The results are represented by mean ± standard deviation. *Activity without ions or inhibitors addition. Different superscript letters represent statistical differences (p < 0.05, n = 3).

Heavy metals constitute a major group of aquatic pollutants and their influence on trypsin activity can be used as a tool for detecting xenobiotics [2,33]. Similar to other tropical fish proteases [1-4,6,10], crevalle jack trypsin showed sensitivity to metal ions, particularly Cd^2+^, Al^3+^, Zn^2+^, Cu^2+^, Pb^2+^, and Hg^2+^ at 1 mM (Table 3). All metal ions tested had a significant effect on the tryptic activity compared to the activity measured in their absence (*p* < 0.05). Presence of Cd^2+^ and Al^3+^ strongly inhibited trypsin activity (>95%), whereas Zn^2+^, Cu^2+^, Pb^2+^, Hg^2+^ were capable of inhibiting the trypsin from 50 - 85%. The effects of Co^2+^, K^+^, Li^+^, Ba^2+^, Mn^2+^, Mg^2+^, and Ca^2+^ were noticeable, but not extreme. Metal ions such as Cd^2+^, Co^2+^, and Hg^2+^ act on sulfhydryl residues in proteins and are responsible for a breakdown of disulfide bonds, generally causing a strong inhibitory effect on enzymatic activity by structural destabilization of the protein [34]. Although Ca^2+^ is reported to be an activator of trypsin in various animals, particularly mammals, such activation was absent in crevalle jack trypsin; in contrast, the enzyme showed decreased activity in the presence of Ca^2+^. Trypsins from other tropical fishes and aquatic organisms also showed similar activity responses to Ca^2+^[1,2,6,35]. These findings point to a possible difference in the structure of the primary calcium-binding site between mammalian pancreatic trypsin and the trypsin from these fish [1]. Cd^2+^, Al^3+^, Zn^2+^, Cu^2+^, and Hg^2+^ (1 mM) also inhibited trypsin obtained from *O*. *niloticus*[1], *A*. *gigas*[2], *D*. *rhombeus*[3], and *P*. *maculatus*[6]. Villalba-Villalba *et al*. [21] reported an inhibitory effect of Hg^2+^ (69%), Mn^2+^ (9%), K^+^ (11%), Mg^2+^ (15%), Li^+^ (16%), Cu^2+^ (29%) at 5mM in vermiculated sailfin catfish trypsin activity. Espósito *et al*. [4] obtained 93.9%, 88.6%, 86.9%, 43.6%, and 42,3% (Cd^2+^, Hg^2+^, Cu^2+^, Zn^2+^, and Al^3+^ at 10 mM, respectively) inhibition of trypsin activity in lane snapper. Values for inhibition of crevalle jack trypsin measured for Cd^2+^, Hg^2+^, Cu^2+^, Zn^2+^, and Al^3+^ (1 mM) were 99.7%, 55.1%, 76.2%, 82.3%, and 99.6%, respectively. According to Freitas-Júnior *et al*. [2], the variation in intensity of inhibition found in the literature using similar ion concentrations is a consequence of species diversity and their adaptations according to the aquatic environment.

### N-terminal amino acid sequence

**Figure 3:**
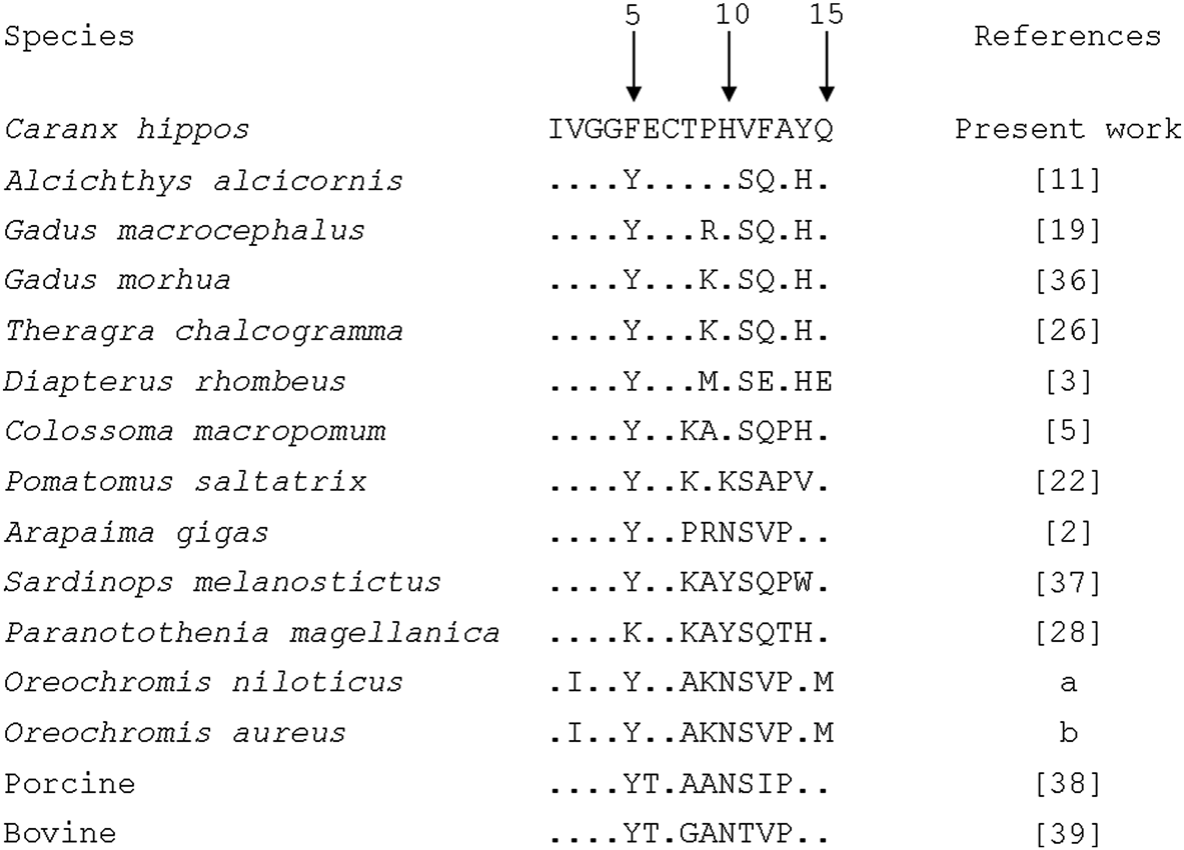
**Comparison between N-terminal amino acid sequences of crevalle jack (*C. hippos*) trypsin and other vertebrates.** The dots represent amino acid residues identical to the query sequence (crevalle jack trypsin) and letters indicate different residues. **a**: GenBank accession number AY510093; **b**: GenBank accession number AY510094.

Fifteen N-terminal amino acids (IVGGFECTPHVFAYQ) of trypsin isolated from *C. hippos* were determined and aligned with the N-terminal sequences of trypsin from other vertebrates [2,3,5,11,19,22,26,28],[36-39] (Figure 3). The first four residues (IVGG) of the sequence are conserved in trypsins from all mammals and most aquatic organisms. Trypsin from *O. niloticus* (GenBank accession number AY510093) and *O. aureus* (AY510094) has an isoleucine residue at position 2. The Cys residue at position 7 is conserved in all trypsins from aquatic organisms and mammals. According to Stroud *et al*. [40], the pancreatic bovine trypsin presents a disulfide bond between the C-7 and C-142 residues. High conservation of the C residue at position 7 in trypsin sequences of several species hints at a structural function of the disulfide bond commonly found in this region. The N-terminal sequence of crevalle jack trypsin revealed a Phe residue at position 5, instead of Tyr, which is a common residue in trypsins from marine animals and mammals. Other trypsins from marine animals also have different residues at position 5, such as trypsin from the Antarctic fish *Paranotothenia magellanica*[28] and the cuttlefish *Sepia officinalis*[41], which have a Lys residue at this position. Although the species investigated in the present study is a subtropical fish, its sequence shows high homology with the fish *A. alcicornis*[11] and other cold-zone fishes including *G. macrocephalus*[19], *G. morhua*[36], and *T. chalcogramma*[26].

## Experimental methods

### Chemicals

All chemicals were of reagent grade and obtained from Sigma Aldrich or Merck. They were used without further purification.

### Enzyme extraction

Fresh crevalle jack specimens (*n* = 3) used in this study measured 74.7 ± 6.32 cm (mean ± SD) in length and were kindly provided by Noronha Pescados LTDA (Recife-PE, Brazil). The pyloric caeca (51.66 ± 3.51 g) were dissected, carefully cleaned using deionized water, and maintained at 4°C during transportation to the laboratory (approximately 30 min). The tissue was homogenized in 0.1 M Tris–HCl pH 8.0 (40 mg tissue/mL buffer) using a tissue homogenizer (4°C) (IKA® RW 20 digital, IKA Works Inc., China). Subsequently, the homogenate was centrifuged (Herolab Unicen MR Centrifuge, Germany) at 10,000 *g* for 10 min at 4°C, and the supernatant (crude extract) was frozen at -20°C and used for further purification steps.

### Enzyme purification

Trypsin was purified following a three-step procedure according Bezerra *et al*. [1] with some modification. Crude extract (100 mL) was incubated at 40°C for 30 min and centrifuged at 10,000 *g* for 10 min at 4°C. The supernatant was collected and fractioned using ammonium sulfate (0%–80% saturation) for 1 h at 4°C. The precipitate exhibiting trypsin activity was then collected using centrifugation and dialyzed with 0.1 M Tris–HCl pH 8.0 (overnight with two buffer changes) at 4°C. A dialyzed sample (5 mg) was applied to a Sephadex^TM^ G-75 (Sigma Chemical Com., St. Louis, MO, USA) column (1.2 × 42 cm) and eluted using 0.1 M Tris–HCl pH 8.0 at a flow rate of 0.34 mL min^-1^ at room temperature. Protein and trypsin activity of each fraction (2 mL) were established according to Warburg and Christian [18] and Freitas-Júnior *et al*. [2], respectively. The protein peaks with highest specific trypsin activity were pooled and used throughout enzyme characterization.

### Sodium dodecylsulfate polyacrylamide gel electrophoresis (SDS-PAGE)

SDS-PAGE was performed according to the Laemmli [42] method using a 4% (w/v) stacking gel and a 12.5% (w/v) separating gel. Molecular mass of the protein bands was estimated using the 198–6.8 kDa molecular mass protein standards (Bio-Rad Laboratories, California, USA).

### Determination of NH2-terminal amino acid sequence

The NH_2_-terminal sequence was determined using the Edman degradation method with a protein sequencer PPSQ-23 (Shimadzu Tokyo, Japan) and an isocratic HPLC system.

### Trypsin activity and protein determination

*p*-nitroaniline release from N-α-benzoyl-_DL_-arginine-p-nitroanilide (BApNA) was followed by an increase in absorbance at 405 nm in a microtiter plate reader (Bio-Rad X-Mark spectrophotometer) as described elsewhere [2]. Controls were assayed without enzyme solution. The absorbance of the samples at 280 nm and 260 nm were measured the the following equation was used to estimate the protein protein content: [protein] mg/mL = 1.5 × A_280 nm_- 0.75 × A_260 nm_[18]. All assays were performed in quadruplicate.

### Physicochemical properties

The influence of temperature and pH on trypsin activity in crevalle jack preparations were studied as follows: the purified enzyme was assayed (in quadruplicate) as described previously at temperatures ranging from 10 to 65°C and pH values from 6.0 to 10.5 (Tris–HCl buffer) using 4 mM BApNA. Thermal stability of the enzyme was assayed at 25°C (in quadruplicate) after pre-incubation for 30 min at temperatures ranging from 30 to 60°C [6].

### Effect of protease inhibitors

Purified crevalle jack trypsin (30 μL) was incubated for 30 min with protease inhibitors (70 μL, 1 mM): PMSF, a serine-protease inhibitor; TLCK, a trypsin-specific inhibitor; benzamidine, a trypsin inhibitor; N-*tosil*-l-*phenylalanine* chloromethyl ketone (TPCK), a chymotrypsin-specific inhibitor; and EDTA, a chelating compound. After incubation, 4 mM BApNA was added and *p*-nitroaniline release was monitored at 405 nm. The enzyme and substrate blank were similarly assayed without enzyme and substrate solution, respectively. Hundred percent values of activities were recorded in the absence of inhibitors. All assays were performed in quadruplicate.

### Kinetic parameters

BApNA was used as a substrate (final concentration from 0.02 mM to 2.4 mM; total volume of 200 μL at 0.1 M Tris–HCl buffer, pH 8.0) in a 96-well microtiter plate. The reaction (in quadruplicate) was initiated by the addition of 30 μL purified enzyme solution (112.5 μg protein/mL) and *p*-nitroaniline release was monitored at 405 nm using a microtiter plate reader. Blanks were similarly prepared without enzyme. Reaction rates were fit into the Michaelis–Menten equation using MicroCal™ Origin™ program version 6.0 (Microcal Software, Inc., MA, USA) [1].

### Effects of metal ions

Samples of purified enzyme (30 μL) were added to a 96-well microtiter plate with a 70 μL 1 mM solution (final concentration) of AlCl_3_, BaCl_2_, CaCl_2_, CdSO_4_, CoCl_2_, CuSO_4_, HgCl_2_, KCl, LiCl, MgCl_2_, MnCl_2_, PbCl_2_, and ZnSO_4_. Deionized water was used to prepare these solutions. After incubation for 30 min, 0.1 M Tris–HCl buffer (70 μL), pH 8.0, and 4 mM BApNA (30 μL) were added. The *p*-nitroaniline produced was measured using a microplate reader at 405 nm after a 30-min reaction. All assays were performed in triplicate.

### Statistical analysis

All values are presented as mean ± standard deviations. The data were analyzed using analysis of variance (ANOVA), followed by a post-hoc Tukey–Kramer test when required. Differences between groups were accepted as significant at 95% confidence level (*p* < 0.05).

## Conclusions

Crevalle jack pyloric caeca trypsin (91.2 U/mg of specific activity and 27.5 kDa) was easily purified approximately 102-fold, recovering about 20% of the enzyme contained in the crude extract. The use of specific substrate, protease inhibitors, and determination of N-terminal amino acid sequence provided additional evidence that a trypsin-like enzyme was obtained. The enzyme showed interesting features such as high activity in alkaline pH, high activity over a wide temperature range, and thermostability. These features indicate the potential of this trypsin for industrial applications.

## Abbreviations

BApNA: N-α-benzoyl-_DL_-arginine-p-nitroanilide; PMSF: Phenylmethylsulphonyl fluoride; TLCK: N-p-*tosyl*-L-*lysine* chloromethyl ketone; TPCK: N-*tosil*-l-*phenylalanine* chloromethyl ketone; EDTA: Ethylenediamine tetraacetic acid; ANOVA: Analysis of variance

## Competing interests

The authors declare that they have no competing interests.

## Authors’ contributions

HMSC, ACVF-J, IPGA, and PMGP performed purification, characterization, and interpretation of the data as well as drafted the manuscript. VO and IYH determined the N-terminal amino acid sequence, interpreted the data, and participated in drafting the manuscript. LBC Jr. and RSB were involved in interpretation of the data, drafting the manuscript, and critically revising it for intellectual content. All authors read and approved the final manuscript.
